# Experimental Investigation on the Bioprotective Role of Trehalose on Glutamine Solutions by Infrared Spectroscopy

**DOI:** 10.3390/ma15124329

**Published:** 2022-06-18

**Authors:** Maria Teresa Caccamo, Salvatore Magazù

**Affiliations:** Department of Mathematical and Computer Sciences, Physical Sciences and Earth Sciences, University of Messina, 98166 Messina, Italy; smagazu@unime.it

**Keywords:** Glutamine, trehalose, bioprotection, thermal response, infrared spectroscopy, trehalose, bioprotection, thermostabilization

## Abstract

Glutamine plays a significant role in several basic metabolic processes and is an important regulator of heat shock protein response. The present work is focused on the analysis of the thermal response of aqueous solutions of Glutamine and aqueous solutions of Glutamine in the presence of Trehalose by means of infrared absorption technique. The performed study shows how in the case of a multicomponent system, characterized by a huge number of spectral contributions whose assignment are questionable, the Spectral Distance (SD) and the Cross Wavelet Correlation (XWT) approaches are able to furnish explanatory parameters that can characterize the variations in the spectra behaviour, which is an efficient tool for quantitative comparisons. With this purpose, the analysis has been performed by evaluating the SD and the XWT parameters for the whole investigated spectral range, i.e., 4000–400 cm^−1^, for scans collected as a function of temperature in the range 20 °C ÷ 60 °C both for Glutamine/Water compounds and for Glutamine /Water/Trehalose mixtures. By means of these analyses, it is found that in aqueous solutions of Glutamine, with respect to aqueous solutions of Glutamine in the presence of Trehalose, the SD and XWT temperature trends follow a linear behaviour where the angular coefficient for Glutamine /Water/Trehalose compounds are lower than that of the Glutamine-Water system in both cases. The obtained findings suggest that Trehalose stabilizes Glutamine against heat treatment.

## 1. Introduction

It is well known that Glutamine, whose chemical formula is C_5_H_10_N_2_O_3_, is a multifunctional amino acid and is the most abundant free amino acid present in the body [1,2,3]. More specifically, immune cells consume Glutamine at a rate similar to or higher than glucose [4,5]. Some specific studies have determined that Glutamine is an essential nutrient for lymphocyte proliferation, cytokine production, and the killing of neutrophil [6,7,8,9]. The metabolic organs, such as the intestine, skeletal muscle, and liver, control both the release and availability of Glutamine into the circulation [10,11,12,13,14,15]. Moreover, Glutamine helps intermediate metabolism in the synthesis of amino sugars and proteins and stimulates insulin secretion from pancreatic beta cells [16,17,18,19,20]. It is useful to stress that Glutamine can diminish the intestinal catabolism of amino acids, which could improve its bioavailability in the systemic circulation [21,22,23]. Glutamine is also a powerful inducer of the heat shock protein response to preserve homeostasis, simplifying repair from damage and cell death [24,25,26,27,28].

**Figure 1:**
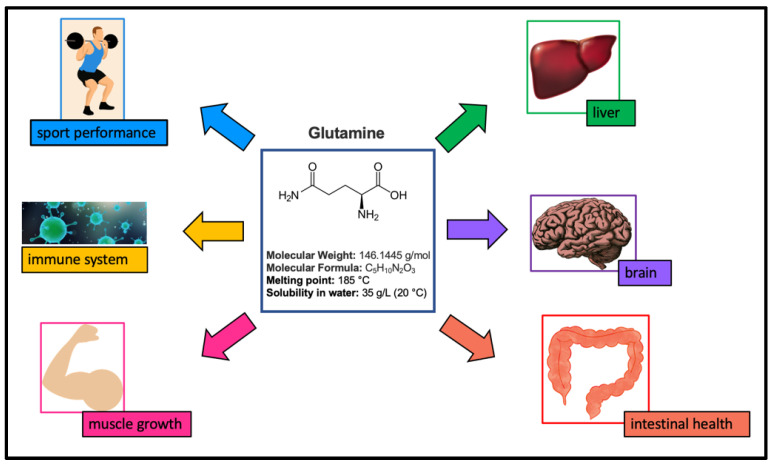
Sketch of some of the physical chemical properties of Glutamine and of its role in the clinical and in the sport fields.

Glutamine is successfully used in both clinical and sport fields. As far as the clinical point of view is concerned, Glutamine constitutes a real, non-toxic transporter of amino groups that are able to cross cell membranes. It enters the bloodstream and reaches the liver, preserves the correct functionality of the immune system, protects the intestinal mucosa from damage induced by chemo and radiotherapy, and can penetrate the blood–brain barrier and enter the brain where it is converted into Glutamate, the most important and widespread excitatory neurotransmitter in the central nervous system. As far as sport is concerned, Glutamine intervenes in increasing the volume of muscle cells, favoring the entry of water into the cells, of amino acids, and of other substances. This activity, according to some research, stimulates protein synthesis, favoring the increase of muscle mass and the improvement of sport performances [29,30]. Figure 1 sketches some of the above reported Glutamine uses, shows its chemical formula, and reports some of its chemical and physical properties.

It should also be stressed that in the cells and tissue of animals, the amino acid is an important compound and is the second most important and abundant compound after water [31,32,33]. In Glutamine, the charge-neutral, polar amino acid has a side chain similar to that of glutamic acid, except the carboxylic acid group is replaced by an amide. Although it is non-essential and conditionally essential in humans, in some instances of stress, the body’s demand for Glutamine increases, and Glutamine must be obtained from the diet. Sugars are widely used as excipients and as structure stabilizers in the formulation of many pharmaceutical products due to the stability conferred to biostructures by the addition of sugars. Two main hypotheses have been proposed to describe the stabilizing effect of sugars on proteins during lyophilization. One suggests that sugars act as a water substituent and that they stabilize biostructures by forming hydrogen bonds at specific sites on the surface of the biostructure. Another hypothesis, referred to as the “vitrification hypothesis”, states that disaccharides form glasses that immobilize biostructures, thereby providing protection against destabilizing processes. The stabilization of biostructures against thermal treatment is referred to as its thermal or structural stability. Spectroscopic techniques [34,35,36,37] have been used to measure the effect of several additives on the thermal stability of biostructures including sugars. Thermal stabilization is important in industries where thermostable enzymes are produced with advantages of longer enzyme shelf life and lowered risk of microbial contamination.

Trehalose is a thermo-stabilizer disaccharide that, despite having the same chemical formula (C_12_H_22_O_11_) of the other two homologous disaccharides, i.e., maltose and sucrose, shows different structures and physical–chemical properties that could account for the higher bioprotectant effectiveness.

The present work is addressed to clarify the role played by Trehalose with specific reference to its bioprotective mechanisms. Specifically, aqueous solutions of Glutamine and aqueous solutions of Glutamine in the presence of Trehalose have been examined as a function of temperature by means of infrared spectroscopy [38,39,40,41].

Generally speaking, many studies have focused on ternary systems such as protein/water/bioprotectant. However, many researchers retain that the protein dynamics are strongly coupled with, and depends on, the solvent properties. Thus, attention has recently also been drawn to water/bioprotectant molecules mixtures, and hence the main interest in investigating binary disaccharide/water systems is due to the fact that the bioprotectant effectiveness on biomolecules are often connected with the thermal properties of the matrix in which the biostructures are immersed.

Several theories have been formulated to explain the function of Trehalose and of its water mixtures. Green and Angell [42] put the bioprotective efficacy in relation to the high glass transition temperature T_g_ of the Trehalose/system water, which leads to a greater amount of water at the transition in respect to the other disaccharides. It should be noted, however, that this hypothesis is not in itself exhaustive since another carbohydrate, the dextran, despite having a higher glass transition temperature than the trehalose, does not have the same efficacy. According to the hypothesis formulated by Crowe [43,44], however, Trehalose would preserve the integrity of the membranes during drying and rehydration, as it can replace water. This hypothesis is supported by the numerical simulations of Grigera [45] who argues that the three-dimensional structure of Trehalose is in perfect structural correspondence with that of water, whose structural and dynamic properties would not be altered by the presence of disaccharide [46,47,48,49,50,51].

## 2. Materials and Experimental Set-Up

Glutamine and Trehalose (99,9 % purity, CAS number 6138-23-4) powders were purchased from Sigma Aldrich Co. (St. Louis, MO, USA). Double distilled water was used for the samples’ preparation. Glutamine/Water compounds (binary system) were prepared by adding to Glutamine double-distilled water (60 wt% Glutamine + 40% H_2_O); for Glutamine/Water/Trehalose mixtures (ternary system) the concentration was the following: 60 wt% Glutamine/40 wt% (Trehalose (25 wt%) + H_2_O (75 wt%)). A buffer system constituted by KH_2_PO_4_/Na_2_HPO_4_ at a concentration value of 0.066 mol/l was used for pH control; in particular, a composition of x_ml_^A^ + (100 − x)_ml_^B^ with A: Na_2_HPO_4_ and B: KH_2_PO_4_, was used. As far as the technique is concerned, Attenuated Total Reflectance Fourier Transform InfraRed (ATR-FTIR) spectroscopy was employed.

Infrared radiation interests the proper energy window where vibrations in molecules are detected. The IR spectrum consists of near (4000–12,800 cm^−1^), mid (200–4000 cm^−1^), and far (10–200 cm^−1^) regions. The mid-IR region is most commonly used for analysis purposes whereas vibrational excitations correspond to changes in the internuclear distances within molecules. Generally, the number of vibrations for a molecule is determined by its degrees of freedom; the latter for most molecules is (3N–6) where N is the number of atoms, while for a linear molecule it is (3N–5). IR spectra are often recorded in reciprocal wavenumbers (cm^−1^). There are certain parts of the mid-IR spectrum that correspond to specific vibrational modes of organic compounds (e.g., 2700–3700 cm^−1^: Hydrogen stretching; 1950–2700 cm^−1^: Triple bond stretching; 1550–1950 cm^−1^: Double bond stretching; 700–1500 cm^−1^: Fingerprint region). An important remark is that when molecules are quite complex, the various vibrational modes become coupled with each other and hence the IR absorption spectral bands can become overlapped and thus complex and difficult to assign. Therefore, although each atomic component has a unique IR spectrum contribution, in the case of multicomponent systems, interpreting IR spectra is not an easy process. In some cases, when using IR spectra for compound identification, the spectrum of the unknown compound can be compared to a library of spectra of known compounds to find a match; however, this procedure is not always possible [52,53,54,55]. For these reasons, we will demonstrate how in the case of a multicomponent system, characterized by a huge number of spectral contributions whose assignment are questionable, the Spectral Distance (SD) and the Cross Wavelet Correlation (XWT) approaches are able to furnish a straightforward parameter, able to characterize the global variations in the spectra behaviour. For our analysis, the Mid-IR range, i.e., 4000 ÷ 400 cm^−1^ has been taken into account. This technique, based on the analysis of absorption spectra, can characterize the vibrational motions and the dynamical and structural properties of the investigated systems [56,57,58,59,60,61]. The investigated samples were heated from 20 °C to 60 °C. The vibrational spectra were registered by the Vertex 70 v spectrometer (Bruker Optics, Ettlingen, Germany) equipped with Platinum diamond ATR, for 64 scans averaging in a spectral range of 4000–400 cm^−1^.

## 3. Methods

In data analysis, model complexity is essentially established by the number of inputs and by the number of employed parameters. As John von Neumann said: “With four parameters I can fit an elephant and with five I can make him wiggle his trunk” [62]. On one hand, overly complex models typically show low bias and high variance, i.e., give rise to an overfitting; in such cases, overfitting constitutes a pitfall since the numerical model fits consider both the relationship between significant variables together and undesirable contributions (e.g., noise). On the other hand, models with a few parameters cannot capture the underlying significant data trend parameters and can have a high bias and a low variance, i.e., give rise to an underfitting. In other words, when model complexity increases, generally bias decreases, and variance increases. Finding a model with the appropriate complexity for a data set requires finding a balance between bias and variance, and hence the choice of a given model complexity is based on the goal of minimizing the total error [63,64,65]. Therefore, distance functions are at the core of important data analysis and processing tools, e.g., PCA, classification, vector median filter, mathematical morphology, and wavelet cross correlation. Distance measures are used to specify the similarity or dissimilarity degree between two data sets, based on specific properties. However, a unique distance measure cannot be defined since its definition depends on the application field, on the descriptive parameters, on the signals under consideration, on the type of similarity (e.g., shape, amplitude, scaling, etc.). Therefore, the selection of distance functions is strongly related to the actual definition of the data to be analyzed. Many of distance functions can be obtained as variations of the Minkowski formula (Equation (1)).

(1) $$d_{q}\left(S_{1},S_{2}\right)={\left(\sum {\left|s_{1,k}-s_{2,k}\right|}^{q}\right)}^{\frac{1}{q}}$$

(2) $$d_{Che}\left(S_{1},S_{2}\right)=\underset{k}{\max}\left(\left|s_{1,k}-s_{2,k}\right|\right)$$

(3) $$d_{RMS}\left(S_{1},S_{2}\right)=\sqrt{\frac{1}{nb}\underset{k}{\sum }{\left(s_{1,k}-s_{2,k}\right)}^{2}}$$

(4) $$d_{\chi_{1}^{2}}\left(S_{1},S_{2}\right)=\underset{k}{\sum }\frac{{\left(s_{1,k}-s_{2,k}\right)}^{2}}{{\left(s_{1,k}+s_{2,k}\right)}^{2}}$$

(5) $$d_{\chi_{2}^{2}}\left(S_{1},S_{2}\right)=\underset{k}{\sum }\frac{{\left(s_{1,k}-s_{2,k}\right)}^{2}}{s_{1,k}+s_{2,k}}$$

where, by varying the Minkowski order parameter q, one obtains the Manhattan distance function for q=1, the Chebyshev distance functions for q=∞, which can be written as Equation (2) and the Euclidean distance function for q=2. Examples of Euclidean distance functions are root mean square error in Equation (3) and the *χ*^2^ distance functions in Equations (4) and (5). This paper uses the Euclidean distance function, and more specifically the so-called spectral distance (SD), to show the advantage of using few explanatory variables for characterizing the spectral features variation in the Fourier Transform absorption intensity as a function of temperature. In particular, the performed study shows how in the case of a multicomponent system, characterized by a huge number of spectral contributions whose assignment are questionable, the SD approach can furnish an explanatory parameter able to characterize the variations in the spectra behaviour and which allows an efficient tool for a quantitative comparison. As far as the baseline correction is concerned, we adopted polynomial baselines methods, which–in contrast to other programs that provide baseline correction methods–are based on least squares fits and can be weighted through particular points, if required. Since a spectral region with many data points has a greater influence on the resulting baseline evaluation than a region with a few data points, an appropriate number of data points in different spectral windows has been chosen. In particular, spectral regions that do not contribute significantly to the baseline were excluded, while the baseline approach was used for the remaining whole spectrum [66,67,68]. Another approach to determine the affinity degree among data sets–in our case, the spectra–is the evaluation of the Wavelet Cross Correlation Coefficient (XWT). To calculate the WXT coefficient it is appropriate to introduce the Wavelet Transform, W(s,t), that compares the signal to shifted and compressed or stretched versions of a wavelet by means of the shift parameter t and of the scale parameter s (s>0), respectively, and the wavelet spectrum WS(s), so the XWT coefficient is calculated by the following formula:

(6) $$XWTC=\frac{\int W_{1}\left(s,t\right)W_{2}^{*}\left(s,t\right)dt}{\sqrt{WS_{1}\left(s\right)WS_{1}\left(s\right)}}$$

where $W_{1}\left(s,t\right)$ is the Wavelet Transform of the first spectrum, $W_{2}^{*}\left(s,t\right)$ is the Wavelet Transform of the second spectrum and * denotes the complex conjugation, while $WS_{1}$ represents the first wavelet spectrum and $WS_{2}$ the second wavelet spectrum. When the value is zero, there is no relationship between the two spectra; when the XWTC is more than 0, the two spectra are correlated. More specifically, if the XWTC is equal to 1 it occurs a positive correlation; if the XWTC is equal to −1, it occurs a negative correlation [69,70,71]. Therefore, it is useful to remember that wavelet analysis has recently found an increasing number of applications in several fields such as image processing, wavevector spectral analyses of neutron scattering, frequency analyses of meteorological time series, financial data sets, medical image technology (e.g., denoising), and more [72,73,74,75,76,77].

## 4. Results and Discussion

**Figure 2:**
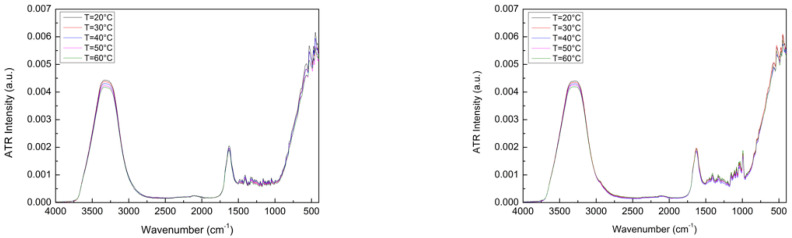
Experimental spectra, in the spectral range of 4000–400 cm^−1^, from 20 °C to 60 °C for: (I) 60 wt% Glutamine + 40% H_2_O (on the **left**); and for (II) 60 wt% Glutamine /40 wt% (Trehalose (25 wt%) + H_2_O (75 wt%)) (on the **right**).

Figure 2 shows experimental spectra for the binary system, i.e., 60 wt% Glutamine + 40% H_2_O, in the spectral range of 4000–400 cm^−1^ from 20 °C to 60 °C on the left and experimental spectra for the ternary system, i.e., 60 wt% Glutamine/40 wt% (Trehalose (25 wt%) + H_2_O (75 wt%)), in the spectral range of 4000–400 cm^−1^ from 20 °C to 60 °C on the right. What emerges from a first glance to these spectra is that the IR band feature changes are more noticeable in the absence of Trehalose. Furthermore, in the presence of Trehalose, there are further multiple O-H and C-O stretching peaks, which are to be attributed to the existence of multiple bonds that give rise to an increase in the spectral complexity.

**Figure 3:**
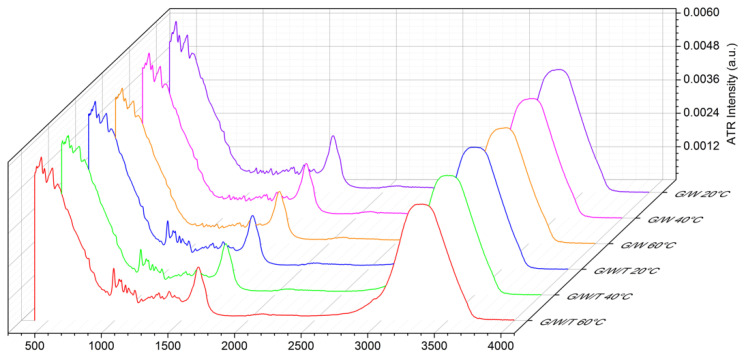
Comparison among the binary (G/W) and the ternary (G/W/T) system spectra for three different temperature values, i.e., T = 20 °C, T = 40 °C, and T = 60 °C.

Figure 3 reports a comparison between the binary and the ternary system of the OH-stretching band for three different temperature values, i.e., T = 20 °C, T = 40 °C, and T = 60 °C.

**Figure 4:**
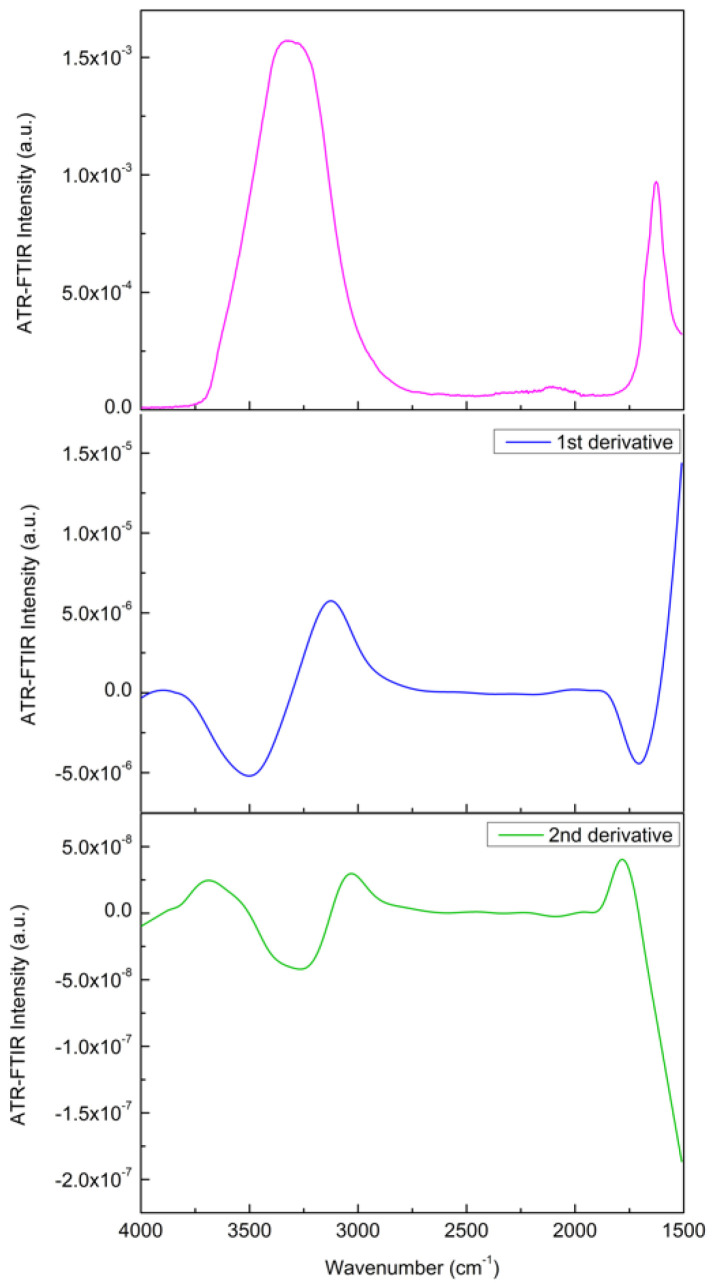
IR spectrum of the Glutamine/Water mixture in the 1500–4000 cm^−1^ spectral range (**top**), together with its first (**center**) and second (**bottom**) order derivatives.

Before investigating the spectra by means of the above-mentioned approaches, as a preliminary analysis, the first derivative and the second derivative of the investigated spectra have been evaluated to accurately determine the peak positions, and the same procedure has been also applied for the deconvoluted component bands [78,79]. Figure 4 reports, as an example, the IR spectrum of the Glutamine/Water mixture in the 1500–4000 cm^−1^ spectral range, together with its first and second order derivatives.

**Figure 5:**
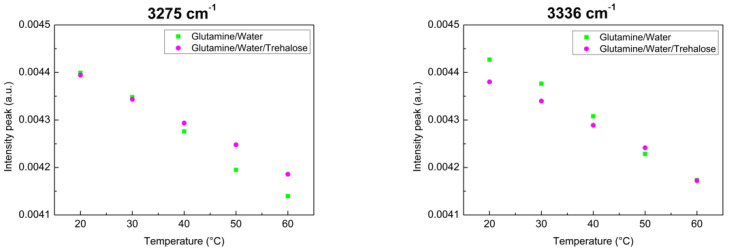
Infrared intensity of Glutamine/Water and of Glutamine/Water/Trehalose mixtures for the subcomponents centered at ω = 3275 cm^−1^ and at ω = 3336 cm^−1^.

To emphasize the thermostability of trehalose, two peaks–in which there are significative changes–have been considered. More precisely, we have evaluated the intensity values for each temperature for the peaks at 3275 cm^−1^ and 3336 cm^−1^. As can be seen from Figure 5, data arrange themselves along a linear behaviour for both the investigated systems, but for the ternary system, the slope value is smaller than that of the binary system for the two peaks. This result suggests that Glutamine in presence of Trehalose is less sensitive to changes in temperature.

**Figure 6:**
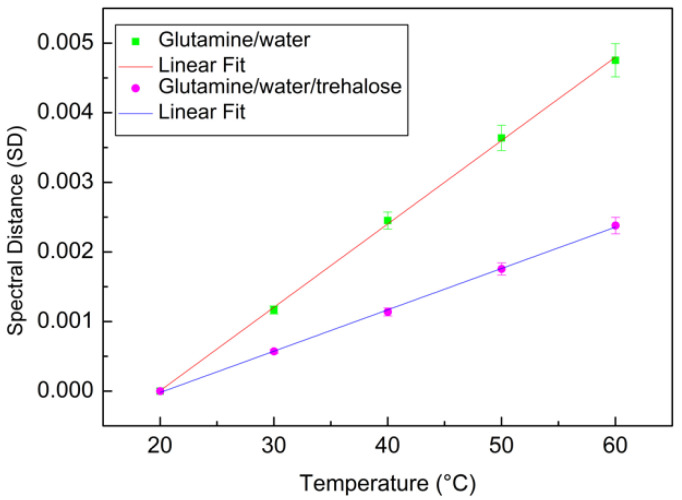
SD as a function of temperature for the binary system (green squares) and for ternary system (magenta circles) together with their linear fits (continuous lines).

Now, in order to characterize the temperature effects as a Spectral Function, we shall consider the Spectral Distance (*SD*) on different frequency scales, i.e., on different spectral ranges:

From a general viewpoint, the Spectral Distance is defined as:

(7) $$SD={\left(\sum {\left[A\left(\omega ,T\right)-A_{i}\left(\omega ,T_{i}\right)\right]}^{2}\cdot \Delta \omega_{res}\right)}^{\frac{1}{2}}$$

where $A_{i}\left(\omega ,T_{i}\right)$ is the absorbance intensity, Δω is the instrumental frequency resolution, and $T_{i}$ is the initial temperature that in the present case is equal to 20 °C. From this evaluation, it is possible to construct a plot that reports the *SD* data versus temperature T [71]. As can be seen from Figure 6, the profiles of the two investigated systems follow a linear trend. To extract quantitative information, a linear model fit has been applied:

(8) y=ax+b

Figure 6 reports the Spectral Distance as a function of temperature for the binary system (green square) and for the ternary system (magenta circle), together with their linear fits (continuous lines).

From this analysis, it is clear that the angular coefficient for the binary system, that is $a=1.19\times 10^{-4}$, is higher in respect to that of the ternary system, that is $a=5.69\times 10^{-5}$.

**Figure 7:**
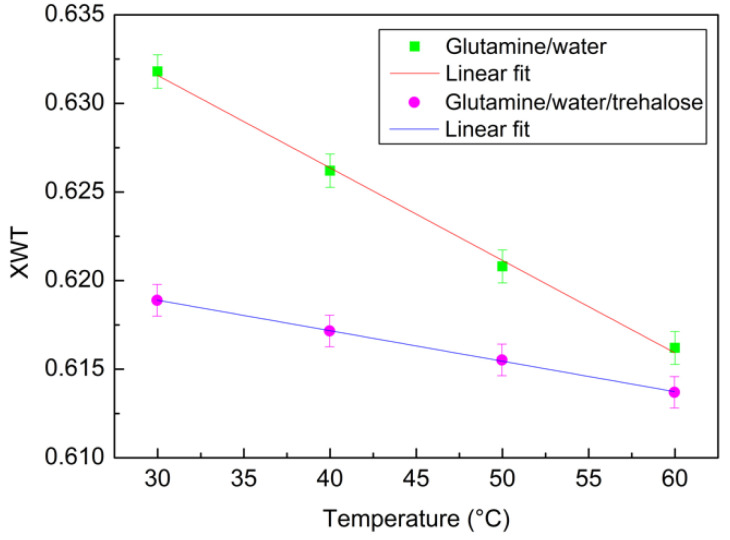
XWT as a function of temperature for the binary system (green squares) and for the ternary system (magenta circles) together with their linear fits (continuous lines).

Another approach to extract quantitative information is the evaluation of the XWT coefficient by means of Equation (6). From this analysis, a linear behaviour in the plot XWT versus temperature can also be seen in Figure 7, which can be fitted by means of Equation (8).

Additionally, in this case, it is clear that the angular coefficient for the binary system, that is $a=-5.44\times 10^{-4}$, is higher in respect to that of the ternary system, which is equal to $a=-1.82\times 10^{-4}$. 

## 5. Conclusions

In this experimental work, the thermal response of aqueous solutions of Glutamine and aqueous solutions of Glutamine in presence of Trehalose by means of infrared absorption were investigated. The data analysis was performed by evaluating the SD and the XWT for the whole investigated spectra, i.e., in a spectral range 4000 ÷ 400 cm^−1^, for scans in temperature in the range from 20 °C to 60 °C. The performed study shows how in the case of a multicomponent system, characterized by a huge number of spectral contributions, the SD and the XWT furnish the same interpretative picture. In particular, the obtained results show that for aqueous solutions of Glutamine, in respect to aqueous solutions of Glutamine in the presence of Trehalose, one obtains a higher value of the SD slope as a function of temperature. More specifically, the temperature trend of such systems follows in both cases a linear behaviour, where the angular coefficient of Trehalose–Glutamine compounds is smaller than that of the aqueous solution of Glutamine. Such findings clearly demonstrate that Trehalose stabilizes Glutamine against thermal stress.

The present work has been addressed to clarify the role played by Trehalose in determining the thermal stabilization of Glutamine. In particular, aqueous solutions of Glutamine and Glutamine aqueous solutions in the presence of Trehalose were examined as a function of temperature by means of Infrared spectroscopy. The data analysis was performed by evaluating the SD and the XWT for the whole investigated spectra, i.e., in a spectral range 4000 ÷ 400 cm^−1^, for scans in temperature in the range from 20 °C to 60 °C. The performed study shows how in the case of a multicomponent system, characterized by a huge number of spectral contributions, the SD and the XWT furnish the same interpretative picture. In particular, the obtained results show that for aqueous solutions of Glutamine, in respect to aqueous solutions of Glutamine in the presence of Trehalose, one obtains a higher value of the SD slope as a function of temperature. More specifically, the temperature trend of such systems follows in both cases a linear behaviour, where the angular coefficient of Glutamine/Water/Trehalose compounds is smaller than that of the aqueous solution of Glutamine. Such findings clearly demonstrate that Trehalose stabilizes Glutamine against thermal stress. What emerges from the present study on Glutamine/Water/Trehalose mixtures performed as a function of temperature is that the thermal stability of the bicomponent Glutamine/Water system increases when Trehalose is added. Such a result confirms that the employment of Trehalose in high added value products is effective and contributes to increase the shelf-life of biomolecules-based products.

## References

[1] Cruzat V., Macedo Rogero M., Noel Keane K., Curi R., Newsholme P. (2018). Glutamine: Metabolism and Immune Function, Supplementation and Clinical Translation. Nutrients.

[2] Stumvoll M., Perriello G., Meyer C., Gerich J. (1999). Role of glutamine in human carbohydrate metabolism in kidney and other tissues. Kidney Int..

[3] Pochini L., Scalise M., Galluccio M., Indiveri C. (2014). Membrane transporters for the special amino acid glutamine: Structure/function relationships and relevance to human health. Front. Chem..

[4] Kedia-Mehta N., Finlay D.K. (2019). Competition for nutrients and its role in controlling immune responses. Nat. Commun..

[5] Festuccia W., Wasinski F., Gregnani M.F., Ornellas F.H., Bacurau A.V.N., Câmara N.O., Araujo R.C., Bacurau R.F. (2014). Lymphocyte Glucose and Glutamine Metabolism as Targets of the Anti-Inflammatory and Immunomodulatory Effects of Exercise. Mediat. Inflam..

[6] Newsholme P. (2001). Why Is L-Glutamine Metabolism Important to Cells of the Immune System in Health, Postinjury, Surgery or Infection?. J. Nutr..

[7] Ardawi M.S.M., Newsholme E.A. (1983). Glutamine metabolism in lymphocytes of the rat. Biochem. J..

[8] Curi R., Williams J.F., Newsholme E.A. (1989). Formation of ketone bodies by resting lymphocytes. Int. J. Biochem..

[9] Rohde T., Maclean D.A., Pedersen B.K. (1996). Glutamine, lymphocyte proliferation and cytokine production. Scand J. Immun..

[10] Newsholme P., Procopio J., Lima M.M., Pithon-Curi T.C., Curi R. (2003). Glutamine and glutamate—Their central role in cell metabolism and function. Cell Biochem. Funct..

[11] Curi R., Lagranha C.J., Doi S.Q., Sellitti D.F., Procopio J., Pithon-Curi T.C. (2005). Glutamine-dependent changes in gene expression and protein activity. Cell Biochem. Funct..

[12] Szondy Z., Newsholme E.A. (1990). The effect of various concentrations of nucleobases, nucleosides or glutamine on the incorporation of [3H]thymidine into DNA in rat mesenteric-lymph-node lymphocytes stimulated by phytohaemagglutinin. Biochem. J..

[13] Wernerman J. (2008). Clinical use of glutamine supplementation. J. Nutr..

[14] Cooney G., Curi R., Mitchelson A., Newsholme P., Simpson M., Newsholme E.A. (1986). Activities of some key enzymes of carbohydrate, ketone body, adenosine and glutamine metabolism in liver, and brown and white adipose tissues of the rat. Biochem. Biophys. Res. Commun..

[15] Parry-Billings M., Dimitriadis G., Leighton B., Bond J., Bevan S., Opara E., Newsholme E. (1990). Effects of hyperthyroidism and hypothyroidism on glutamine metabolism by skeletal muscle of the rat. Biochem. J..

[16] Carvalho-Peixoto J., Alves R.C., Cameron L.-C. (2007). Glutamine and carbohydrate supplements reduce ammonemia increase during endurance field exercise. Appl. Phys. Nutr. Metab..

[17] De Oliveira D.C., da Silva Lima F., Sartori T., Santos A.C.A., Rogero M.M., Fock R.A. (2016). Glutamine metabolism and its effects on immune response: Molecular mechanism and gene expression. Nutrire.

[18] Newsholme P., Lima M., Procopio J., Pithon-Curi T., Doi S., Bazotte R., Curi R. (2003). Glutamine and glutamate as vital metabolites. Braz. J. Med. Biol. Res..

[19] Roth E., Oehler R., Manhart N., Exner R., Wessner B., Strasser E.-M., Spittler A. (2002). Regulative potential of glutamine—Relation to glutathione metabolism. Nutrition.

[20] Roth E. (2008). Nonnutritive effects of glutamine. J. Nutr..

[21] Kim M.H., Kim H. (2017). The Roles of Glutamine in the Intestine and Its Implication in Intestinal Diseases. Int. J. Mol. Sci..

[22] Boelens P.G., Nijveldt R.J., Houdijk A.P.J., Meijer S., van Leeuwen P.A.M. (2001). Glutamine Alimentation in Catabolic State. J. Nutrition..

[23] Dai Z.L., Li X.L., Xi P.B., Zhang J., Wu G., Zhu W.Y. (2013). L-Glutamine regulates amino acid utilization by intestinal bacteria. Amino Acids.

[24] Wu L., Tang Z., Chen H., Ren Z., Ding Q., Liang K., Sun Z. (2021). Mutual interaction between gut microbiota and protein/amino acid metabolism for host mucosal immunity and health. Anim. Nutr..

[25] Senf S.M., Howard T.M., Ahn B., Ferreira L.F., Judge A.R. (2013). Loss of the inducible Hsp70 delays the inflammatory response to skeletal muscle injury and severely impairs muscle regeneration. PLoS ONE.

[26] Senf S.M. (2013). Skeletal muscle heat shock protein 70: Diverse functions and therapeutic potential for wasting disorders. Front. Physiol..

[27] Sottile M.L., Nadin S.B. (2018). Heat shock proteins and DNA repair mechanisms: An updated overview. Cell Stress Chaperones.

[28] van Noort J.M., Bugiani M., Amor S. (2017). Heat Shock Proteins: Old and Novel Roles in Neurodegenerative Diseases in the Central Nervous System. CNS Neurol. Disord. Drug Targets.

[29] Ramezani A.A., Rayyani E., Bahreini M., Mansoori A. (2019). The effect of glutamine supplementation on athletic performance, body composition, and immune function: A systematic review and a meta-analysis of clinical trials. Clin. Nutr..

[30] Coqueiro A.Y., Rogero M.M., Tirapegui J. (2019). Glutamine as an Anti-Fatigue Amino Acid in Sports Nutrition. Nutrients.

[31] Labow B.I., Souba W.W., Abcouwer S.F. (2001). Mechanisms governing the expression of the enzymes of glutamine metabolism—Glutaminase and glutamine synthetase. J. Nutr..

[32] Berg A., Norberg Å., Martling C.-R., Gamrin L., Rooyackers O., Wernerman J. (2007). Glutamine kinetics during intravenous glutamine supplementation in ICU patients on continuous renal replacement therapy. Intensiv. Care Med..

[33] Eagle H., Oyama V.I., Levy M., Horton C.L., Fleischman R. (1956). The growth response of mammalian cells in tissue culture to L-glutamine and L-glutamic acid. J. Biol. Chem..

[34] Zhou C., Guo X., Wang S., Zhu Y., Mu D. (2011). Effects of temperature and additives on stability and spectrum of a therapeutic fibroblast growth factor. DARU J. Pharm. Sci..

[35] Santos M.I., Gerbino E., Tymczyszyn E., Gomez-Zavaglia A. (2015). Applications of Infrared and Raman Spectroscopies to Probiotic Investigation. Foods.

[36] Kozak S., Lercher L., Karanth M.N., Meijers R., Carlomagno T., Boivin S. (2016). Optimization of protein samples for NMR using thermal shift assays. J. Biomol. NMR.

[37] Wang R., Xu S., Yue Y., Wang X. (2020). Thermal behavior of materials in laser-assisted extreme manufacturing: Raman-based novel characterization 2020. Int. J. Extrem. Manuf..

[38] Magazù S., Calabrò E., Caccamo M.T. (2018). Experimental study of thermal restraint in bio-protectant disaccharides by FTIR spectroscopy. Open Biotechn. J..

[39] de Almeida F.S., de Andrade Silva C.A., Lima S.M., Suarez Y.R., da Cunha Andrade L.H. (2018). Use of Fourier transform infrared spectroscopy to monitor sugars in the beer mashing process. Food Chem..

[40] Wright W.W., Guffanti G.T., Vanderkooi J.M. (2003). Protein in Sugar Films and in Glycerol/Water as Examined by Infrared Spectroscopy and by the Fluorescence and Phosphorescence of Tryptophan. Biophys. J..

[41] Caccamo M.T., Magazù S. (2016). Tagging the oligomer-to-polymer crossover on EG and PEGs by infrared and Raman spectroscopies and by wavelet cross-correlation spectral analysis. Vib. Spectrosc..

[42] Green J.L., Angell C.A. (1989). Phase relations and vitrification in saccharide-water solutions and the trehalose anomaly. J. Phys. Chem..

[43] Crowe J.H., Carpenter J.F., Crowe L.M. (1998). The role of vitrification in anhydrobiosis. Ann. Rev. Physiol..

[44] Crowe J.H., Crowe L.M., Oliver A.E., Tsvetkova N., Wolkers W., Tablin F. (2001). The trehalose myth revisited: Introduction to a symposium on stabilization of cells in the dry state. Cryobiology.

[45] Donnamaria M.C., Howard E.I., Grigera J.R. (1994). Interaction of water with α,α-trehalose in solution: Molecular dynamics simulation approach. J. Chem. Soc. Faraday Trans..

[46] Ball C.D., Hardt D.T., Duddles W.J. (1943). The influence of sugars on the formation of sulfhydryl groups in heat denaturation and heat coagulationof egg albumin. J. Biol. Chem..

[47] Magazù S., Migliardo F., Affouard F., Descamps M., Telling M.T.F. (2010). Study of the Relaxational and Vibrational Dynamics of Bioprotectant Glass-Forming Mixtures by Neutron Scattering and Molecular Dynamics Simulation. J. Chem. Phys..

[48] Lombardo D., Calandra P., Caccamo M.T., Magazuù S., Kiselev M.A. (2019). Colloidal stability of liposomes. AIMS Mat. Sci..

[49] Fenimore P.W., Frauenfelder H., Magazù S., McMahon B.H., Mezei F., Migliardo F., Young R.D., Stroe I. (2013). Concepts and problems in protein dynamics. Chem. Phys..

[50] Caccamo M.T., Gugliandolo C., Zammuto V., Magazù S. (2020). Thermal properties of an exopolysaccharide produced by a marine thermotolerant Bacillus licheniformis by ATR-FTIR spectroscopy. Int. J. Biol. Macrom..

[51] Maisano G., Majolino D., Migliardo P., Venuto S., Aliotta F., Magazù S. (1993). Sound Velocity and Hydration Phenomena in Aqueous Polymeric Solutions. Mol. Phys..

[52] Tsai S.R., Hamblin M.R. (2017). Biological effects and medical applications of infrared radiation. J. Photochem. Photobiol. B.

[53] Timchenko S.D., Dement’ev V.A. (1993). Classification of IR spectra of organic compounds by the methods of multidimensional statistics. J. Struct. Chem..

[54] Hemen D., Lalita L., Nisha C., Purvi K., Bhakti D., Sudhir N. (2013). Surface Modification of Polyester Fabric by Non- Thermal Plasma Treatment and Its Effect on Coloration Using Natural Dye. J. Pol. Mat..

[55] Schwanninger M., Rodrigues J.C., Fackler K.A. (2011). Review of Band Assignments in near Infrared Spectra of Wood and Wood Components. J. Near Infrared Spectrosc..

[56] Tatulian S.A. (2019). FTIR Analysis of Proteins and Protein-Membrane Interactions. Methods Mol. Biol..

[57] Shai Y. (2013). ATR-FTIR studies in pore forming and membrane induced fusion peptides. Biochim. Biophys Acta.

[58] Vigano C., Manciu L., Buyse F., Goormaghtigh E., Ruysschaert J.M. (2000). Attenuated total reflection IR spectroscopy as a tool to investigate the structure, orientation and tertiary structure changes in peptides and membrane proteins. Biopolymes.

[59] Fahmy K. (2001). Application of ATR-FTIR spectroscopy for studies of biomolecular interactions. Recent Res. Dev. Chem..

[60] Wharton C.W. (2000). Infrared spectroscopy of enzyme reaction intermediates. Nat. Prod. Rep..

[61] Barth A., Zscherp C. (2002). What vibrations tell us about proteins?. Quart. Rev. Biophys.

[62] Mayer J. (2010). Drawing an elephant with four complex parameters. Am. J. Phys..

[63] Pols L.C.W. (1971). Real-time recognition of spoken words. IEEE Trans. Comp..

[64] Pols L.C.W., Van Der Kamp L.J., Plomp R. (1969). Perceptual and Physical Space of Vowel Sounds. J. Acoust. Soc. Am..

[65] Konukoglu E., Glocker B., Criminisi A., Pohl K.M. (2013). WESD—Weighted Spectral Distance for Measuring Shape Dissimilarity. IEEE Trans. Pattern Anal. Mach. Intell..

[66] Hilda D., Noël R., Hardeberg J. (2015). A Comprehensive Evaluation of Spectral Distance Functions and Metrics for Hyperspectral Image Processing. IEEE J. Sel. Top. Appl. Earth Obs. Remote Sens..

[67] Zhang F., Tang X., Tong A., Wang B., Wang J. (2020). An Automatic Baseline Correction Method Based on the Penalized Least Squares Method. Sensors.

[68] Liland K., Almøy T., Bjørn-Helge M. (2010). Optimal Choice of Baseline Correction for Multivariate Calibration of Spectra. Appl. Spectrosc..

[69] Li H., Nozaki T. (1997). Wavelet cross-correlation analysis and its application to a plane turbulent jet. JSME Int. J. Ser. B Fluids Therm. Eng..

[70] Grinsted A., Moore J.C., Jevrejeva S. (2004). Application of the cross wavelet transform and wavelet coherence to geophysical time series. Nonlinear Process. Geophys..

[71] Torrence C., Compo G. (1998). A Practical Guide to Wavelet Analysis. Bull. Am. Meteorol. Soc..

[72] Caccamo M.T., Calabrò E., Cannuli A., Magazù S. (2016). Wavelet Study of Meteorological Data Collected by Arduino-Weather Station: Impact on Solar Energy Collection Technology. MATEC Web of Conferences.

[73] Ramsey J.B. (1999). The Contribution of Wavelets to the Analysis of Economic and Financial Data. Philos. Trans. R. Soc. Lond. Ser. A Math. Phys. Eng. Sci..

[74] Georgieva V., Plamen P., Zlatareva D. (2021). Medical image processing based on multidimensional wavelet transforms—Advantages and trends. AIP Conference Proceedings.

[75] Addison P.S. (2005). Wavelet transforms and the ECG: A review. Physiolog. Meas..

[76] Bnou K., Raghay S., Hakim A. (2020). A wavelet denoising approach based on unsupervised learning model. EURASIP J. Adv. Signal Process..

[77] Giuffrida S., Cottone G., Librizzi F., Cordone L. (2003). Coupling between the thermal evolution of the heme pocket and the external matrix structure in trehalose coated carboxymyoglobin. J. Phys. Chem. B.

[78] Rieppo L., Saarakkala S., Närhi T., Helminen H.J., Jurvelin J.S., Rieppo J. (2012). Application of second derivative spectroscopy for increasing molecular specificity of Fourier transform infrared spectroscopic imaging of articular cartilage. Osteoart. Cartil..

[79] Fahelelbom K.M.S., Saleh A., Mansour R., Sayed S. (2020). First derivative ATR-FTIR spectroscopic method as a green tool for the quantitative determination of diclofenac sodium tablets. F1000Research.

